# *Vibrio aestuarianus* clade A and clade B isolates are associated with Pacific oyster (*Magallana gigas*) disease outbreaks across Ireland

**DOI:** 10.1099/mgen.0.001078

**Published:** 2023-08-04

**Authors:** Nicola M. Coyle, Ciar O'Toole, Jennifer C. L. Thomas, David Ryder, Edward J. Feil, Michelle Geary, Timothy P. Bean, Andrew Wokorac Joseph, Ava Waine, Deborah Cheslett, David W. Verner-Jeffreys

**Affiliations:** ^1^​ Centre for Environment Fisheries and Aquaculture, Weymouth DT4 8UB, UK; ^2^​ The Milner Centre for Evolution, Department of Life Sciences, University of Bath, Bath BA2 7AY, UK; ^3^​ Marine Institute, Oranmore, Co. Galway H91 R673, Ireland; ^4^​ The Roslin Institute, The University of Edinburgh, Easter Bush Campus, Midlothian EH25 9RG, UK; ^5^​ Newcastle University, School of Natural and Environmental Sciences, Newcastle Upon Tyne, NE1 7RU, UK

**Keywords:** *Vibrio aestuarianus*, *Vibrio splendidus*, summer mortality syndrome, Ireland, aquaculture, transmission

## Abstract

Bacteria from the family *Vibrionaceae* have been implicated in mass mortalities of farmed Pacific oysters (*Magallana gigas*) in multiple countries, leading to substantial impairment of growth in the sector. In Ireland there has been concern that *Vibrio* have been involved in serious summer outbreaks. There is evidence that *Vibrio aestuarianus* is increasingly becoming the main pathogen of concern for the Pacific oyster industry in Ireland. While bacteria belonging to the *Vibrio splendidus* clade are also detected frequently in mortality episodes, their role in the outbreaks of summer mortality is not well understood. To identify and characterize strains involved in these outbreaks, 43 *Vibrio* isolates were recovered from Pacific oyster summer mass mortality episodes in Ireland from 2008 to 2015 and these were whole-genome sequenced. Among these, 25 were found to be *V. aestuarianus* (implicated in disease) and 18 were members of the *V. splendidus* species complex (role in disease undetermined). Two distinct clades of *V. aestuarianus* – clade A and clade B – were found that had previously been described as circulating within French oyster culture. The high degree of similarity between the Irish and French *V. aestuarianus* isolates points to translocation of the pathogen between Europe’s two major oyster-producing countries, probably via trade in spat and other age classes. *V. splendidus* isolates were more diverse, but the data reveal a single clone of this species that has spread across oyster farms in Ireland. This underscores that *Vibrio* could be transmitted readily across oyster farms. The presence of *V. aestuarianus* clades A and B in not only France but also Ireland adds weight to growing concern that this pathogen is spreading and impacting Pacific oyster production within Europe.

## Outcome

Pacific oyster culture in Ireland has increasingly suffered from summer mass mortality events. Many of these mortalities in recent years have been associated with Vibrio aestuarianus; the role of another pathogen, Vibrio splendidus has, so far, remained inconclusive. Here we show that two clades of V. aestuarianus are circulating in Ireland, and that these are members of two clades that have previously caused extensive oyster die offs in France. Their discovery in Ireland is consistent with transport of infected oyster stock between the two countries. Although V. splendidus-like strains in Ireland were highly diverse, a small clonal group was detected that appears to have spread rapidly from a single source to disparate locations in Ireland. Combined, these findings highlight the appearance of a highly pathogenic Vibrio in Ireland, and the risk of transmission between interconnected oyster production industries in Europe.

## Data Summary

Sequences generated in this study were deposited in the National Center for Biotechnology Information (NCBI) database. Accession number: PRJNA797364. Publicly accessed genomes are listed in Table S2. This article contains data hosted by Microreact.

## Introduction

While the aquaculture industry has expanded rapidly in the past 50 years, oyster production has struggled to keep pace with other aquaculture products [1]. One of the significant factors constraining the development of oyster aquaculture has been infectious disease [1, 2]. Pacific oysters (*Magallana gigas*, formerly *Crassostrea gigas*) are an important farmed species [3, 4], with 620 000 tonnes produced on average each year worldwide between 2010 to 2019, worth an estimated USD $1.29 billion a year [5]. France is the major European producer (84 760 tonnes in 2019), although there are significant industries in other European countries, including Ireland (10 460 tonnes in 2019). In France and elsewhere, there have been increased reports of disease outbreaks responsible for the depletion of oyster stocks over the last decade [3]. These present major socioeconomic consequences for the future of the oyster farming industry [6].

Episodes of abnormal mortality of Pacific oysters affecting all age classes have been described globally since the 1950s. Mortality of larvae and spat has been linked to the presence of a number of pathogenic agents, including ostreid herpes virus 1 (OsHV-1), whilst the term summer mortality syndrome has been coined to describe those events of mixed aetiology in the summer months affecting older oysters where gonad maturation is present [7]. Studies have shown that the causes of summer mortality syndrome are complex, often involving a combination of physiological and environmental stress, alongside the presence of pathogens [8], particularly bacteria belonging to the genus *Vibrio *, including *V. aestuarianus* and *V. splendidus* [9].

In the summer of 2008, abnormally high mortality episodes affecting spat and juvenile Pacific oysters were reported in both France and Ireland. The losses were linked to the emergence of a new variant of OsHV-1, termed ostreid herpes virus 1 µVariant (OsHV-1 µVar) [10]. Both *V. splendidus* and *V. aestuarianus* were also detected during a number of these events, although their role in these events was never fully elucidated [7]. Between 2011 and 2013, a new mortality phenomenon began to emerge in France affecting principally adult Pacific oysters. During this period, the frequency of detection of *V. aestuarianus* in cases of adult mortality increased significantly from 30 % in 2011 to 77 % of cases in 2013, becoming the principle pathogen detected during summer mortality episodes in adult oysters in France [11].

The Pacific oyster industry in Ireland is heavily dependent on the importation of spat, which is predominantly sourced from France [12]. Hence, following the reports of increased detections of *V. aestuarianus* in cases of adult mortality in France, a monitoring programme and a retrospective study were instigated in Ireland to determine the extent of its distribution in Ireland. In this study, we characterize and compare 43 *Vibrio* isolates recovered from diseased Irish oysters from 2008 to 2015 using whole-genome sequencing.

We show, firstly, that a high proportion of these oyster die-offs are associated with the presence of *V. aestuarianus* isolates from two oyster-associated *V. aestuarianus* subsp. *francensis* clades, clade A and clade B previously shown to be a major cause of summer mortality syndrome in France [11]. Secondly, we showcase differences in gene content diversity in these clades. Thirdly we show that *V. splendidus* strains present in Irish oysters are diverse, but a small clonal group was detected in 2009 in multiple locations.

## Methods

### Bacterial isolation and initial characterization

**Fig. 1.**
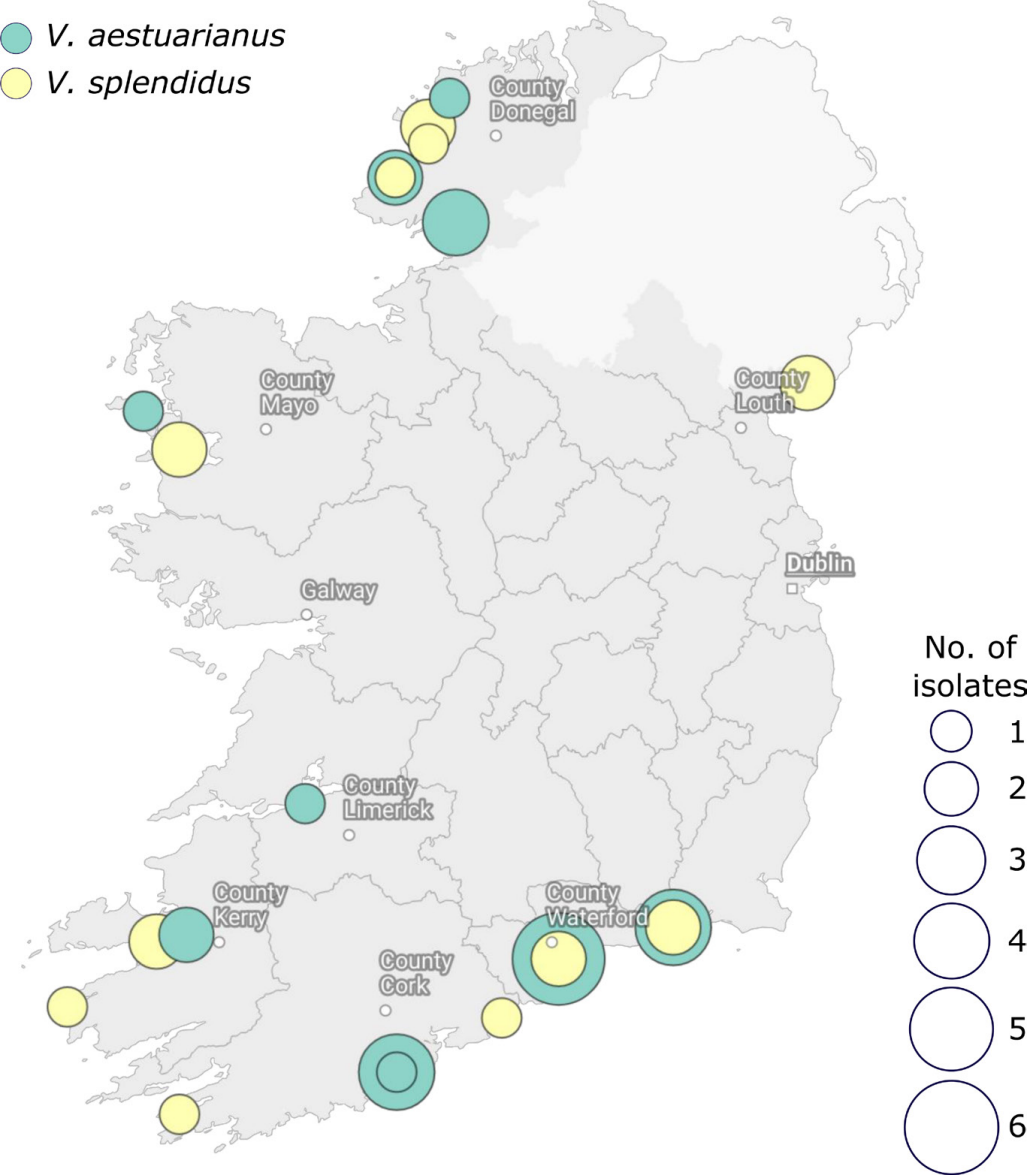
Map of 43 strains sampled across Ireland; 24 *V *. *splendidus* and 18 *V *. *aestuarianus* isolates were collected from 23 locations. Pie charts indicate the proportion of each species sequenced from each location. These nodes are weighted by the number of isolates (scale, 1–6).

Forty-three *Vibrio* isolates obtained from oysters of varying age classes (Fig. 1 and Table 1) were collected from 22 sites around the Republic of Ireland between 2008 and 2015. Isolates were recovered from either haemolymph or crushed gill tissues and characterized. In most cases, isolates were recovered from sites where there were significant ongoing mortalities taking place (Table 1). They were then stored at −80 °C on cryovials using the protect storage system following the manufacturer’s instructions (Technical Service Consultants Ltd). To identify *V. aestuarianus* strains, primers developed for *V. aestuarianus* identification were used as described by McCleary and Henshilwood [13]. A 16S analysis was used to identify other *Vibrio* species as described by Lane *et. al* [14]. Primers used here were forward primer: 5′-AGAGTTTGATCCTGGCTCAG, reverse primer: 5′-GWATTACCGCGGCKGCTG.

**Table 1.** *
Vibrio* strains selected for sequencing from Irish oysters

ID	Year of extraction	Site of extraction	Tissue sample was extracted from	Reported mortality rate %	Age class	Species
16 025	2009	Loughros Beag	Gill	80–90	0+, 1+	* V. aestuarianus *
16 028	2014	Woodstown	Haemolymph	60–70	3+	* V. aestuarianus *
16 030	2014	Kinsale	Haemolymph	90	2+	* V. aestuarianus *
16 033	2014	Dungarvan	Gill	10–20	0+, 1+	* V. aestuarianus *
16 034	2014	Achill	Gill	10	0+	* V. aestuarianus *
16 036	2015	Castlemaine	Haemolymph	40	2+	* V. aestuarianus *
16 041	2015	Woodstown	Gill	1	1+	* V. aestuarianus *
16 043	2015	Donegal Bay	Haemolymph	50–90	2+	* V. aestuarianus *
16 044	2015	Dungarvan	Haemolymph	15–50	2+	* V. aestuarianus *
16 048	2015	Dungarvan	Haemolymph	1	2+	* V. aestuarianus *
16 049	2015	Dungarvan	Haemolymph	1.5	2+	* V. aestuarianus *
16 050	2015	Woodstown	Gill	92	0+	* V. aestuarianus *
16 053	2015	Kinsale	Haemolymph	70–80	2+	* V. aestuarianus *
16 054	2010	Poularone Creek	Gill	30–40	0+	* V. aestuarianus *
16 056	2015	Donegal Bay	Haemolymph	20	2+	* V. aestuarianus *
16 057	2015	Dungarvan	Haemolymph	5.5	2+	* V. aestuarianus *
16 058	2015	Dungarvan	Haemolymph	5	2+	* V. aestuarianus *
16 059	2013	Kinsale	Gill	15–20	1+	* V. aestuarianus *
16 060	2015	Donegal Bay	Haemolymph	2	2+	* V. aestuarianus *
16 062	2015	Gweedore	Haemolymph	43	1+	* V. aestuarianus *
16 063	2014	Kinsale	Haemolymph	90	1+	* V. aestuarianus *
16 066	2013	Oysterhaven	Gill	50	0+, 1+, 2+	* V. aestuarianus *
16 067	2015	Woodstown	Haemolymph	0	2+	* V. aestuarianus *
16 070	2014	Woodstown	Haemolymph	60–70	1+	* V. splendidus* sensu stricto
16 071	2014	Castlemaine	Haemolymph	30	2+	* V. aestuarianus *
16 029	2009	Ballymacoda Bay	Haemolymph	20	0+	* V. splendidus* sensu stricto
16 035	2009	Clew Bay	Haemolymph	10	0+	* V. splendidus* sensu stricto
16 037	2013	Carlingford Lough	Gill	50	0+	* V. splendidus* sensu stricto
16 051	2009	Clew Bay	Haemolymph	75	0+	* V. splendidus* sensu stricto
16 052	2013	Dungloe Bay	Haemolymph	20	1+	* V. splendidus* sensu stricto
16 065	2009	Dungloe Bay	Haemolymph	35	0+	* V. splendidus* sensu stricto
16 069	2014	Woodstown Strand	Haemolymph	60	3+	* V. splendidus* sensu stricto
16 072	2009	Clew Bay	Haemolymph	3–50 %	0+	* V. splendidus* sensu stricto
16 073	2009	Valentia River	Haemolymph	45	0+	* V. splendidus* sensu stricto
16 074	2008	Dungarvan Harbour	Haemolymph	15	1+	* V. splendidus* sensu stricto
16 061	2008	Dungarvan Harbour	Haemolymph	15	1+	* V. splendidus* sensu stricto
16 077	2016	Woodstown Strand	Haemolymph	25	3+	* V. splendidus* sensu stricto
16 078	2008	Castlemaine Harbour	Haemolymph	85	1+	* V. splendidus* sensu stricto
16 079	2015	Trawenagh Bay	Haemolymph	70	1+	* V. splendidus* sensu stricto
16 040	2009	Loughros Beag	Haemolymph	10	1+	* V. splendidus* sensu lato
16 042	2013	Dunmanus Bay	Haemolymph	40	1+	* V. splendidus* sensu lato
16 075	2010	Carlingford Lough	Haemolymph	30	0+	* V. splendidus* sensu lato

### DNA extraction and quantification

DNA was extracted from the isolates using the MasterPure Gram-positive DNA extraction kit (cat. no. MGP04100; Epicentre). The standard protocol was modified slightly to accommodate for the isolates being Gram-negative organisms. In summary, a 1 µl loopful of bacteria [previously sub-cultured onto seawater agar (SWA)] was placed into a 1.5 ml Eppendorf tube containing 1 ml 0.9 % saline. The solution was centrifuged at 1500 r.p.m., supernatant was removed and 150 µl TE buffer was added. Samples were vortexed to resuspend the pellet and 150 µl of a premade dilution of proteinase K in Gram-positive lysis solution was added to each sample, at a concentration of 1 µl proteinase K 150 µl^−1^ of Gram-positive lysis solution. The samples were vortexed and subsequently incubated at 65–70 °C for 15 min, which included vortexing every 5 min. Samples were cooled to 37 °C and then put on ice for 3–5 min, following which 175 µl of MPC protein precipitation reagent was added to each sample. Samples were vortexed and centrifuged at 1500 r.p.m. and 4 °C for 10 min. The supernatant was collected (pellets discarded) and 500 µl of isopropanol was added and samples were inverted 30–40 times and centrifuged again at 1500 r.p.m. and 4 °C for 10 min. The supernatant was removed, 70 % ethanol was added, and samples were centrifuged for a final time at 1500 r.p.m. and 4 °C for 5 min. Finally, the supernatant was removed, and samples were resuspended in 100 µl of molecular grade water and stored at −80 °C until future use. The extracted DNA was quantified using a Quantus fluorometer (Promega), and quality assessed using a NanoDrop ND-1000 Spectrophotometer (Thermo). Only those samples that passed the quality check were selected for high-throughput (Illumina) sequencing.

### Illumina sequencing

Isolates were sequenced using an Illumina Miseq according to the standard protocols produced by the manufacturer. In brief, the DNA quantities were checked by fluorescence, diluted and prepared for sequencing with the Illumina Nextera XT library preparation kit, including optional 96-barcode adapters. Cleaned libraries were then sized-checked with an Agilent Technology 2100 Bioanalyzer using a high-sensitivity DNA chip and quantified by a Promega Quantus fluorometer using a OneDNA protocol. Finally, libraries were normalized, pooled and sequenced on the Miseq with Illumina V3 600 chemistry.

### Quality check

Sequences were trimmed using Trimmomatic version 0.36, with the parameters :ILLUMINACLIP:*:2 : 30 : 10 MINLEN:36 SLIDINGWINDOW:4 : 20 TOPHRED64 [15]. FastQC version 0.11.7 was used to check the quality of trimmed reads, and to ensure that there were no significant contaminants [16].

### Assembly and identification of open reading frames

Spades version 3.13.1 was used for assembly, with the parameters: -k 55,77,87,99,107,117,127 – careful –only assembler [17]. Prior to assembly, reads were downsampled to 100× coverage where needed. Reads were also merged using Flash v 1.2.11 with a minimum overlap of 10 bp, and maximum overlap equal to the maximum length of the reads per sample [18]. Contigs <500 bp were removed. In order to remove contigs with low coverage, reads were mapped to the assembly using bwa and SAMtools v1.8 was used to calculate coverage [19, 20]. Contigs with <10 % of the overall genome coverage, or at minimum 5× coverage, were removed. Assembled genomes were annotated using version 1.13 of Prokka, with the options: –addgenes –centre XXX –mincontiglen 200 –cdsrnaolap [21]. Quality assessment of assemblies was carried out using QUAST v4.6.3 [22]. QC scores for all reads and assemblies are provided in Table S1 (available in the online version of this article).

### Accessing public genomes of *V. splendidus* and *V. aestuarianus*

We obtained publicly available WGS data for *V. splendidus* and *V. aestuarianus* in order to place the isolates from Irish oysters into broader phylogenetic contexts. Thirteen *V. aestuarianus* genomes were contributed by Goudenège *et al*. [23]. Assembled genomes of 102 isolates previously characterized as *V. splendidus* were downloaded from the National Center for Biotechnology Information (NCBI) database [24]. Information on each of these isolates can be found in Table S2. All subsequent genomic analysis was performed using datasets of 38 *V *. *aestuarianus* and 120 *V *. *splendidus* genomes.

### Pangenome construction

A comprehensive pangenome of each species was constructed for using PIRATE [25], a toolbox for bacterial pangenomics analysis. Briefly, PIRATE produces putative gene families by first clustering protein-coding sequences using CD-HIT and carrying out an all-versus-all alignment of representative sequences for each cluster using Diamond’s blastp-like algorithm, filtering results that fall below a given threshold, and using the remaining bit scores to identify putative ‘gene families’ using the MCL algorithm. The process is then repeated, progressively filtering lower scoring matches, to identify the highest threshold at which genes are classified as belonging to a single family, following which there are some additional steps to identify orthologues, paralogues and fission loci. We used Phandango version 1.3.0 [26] to visualize the distribution of gene families within each population. Concatenated core genome alignments were built using PIRATE with default parameters. Individual gene family alignments were calculated using MAFFT for gene families present in at least 95 % of isolates with a maximum genome dosage (copy number) of 1.25, before being concatenated into a core genome alignment [27]. We used R version 3.2.3 [28] for statistical analysis and data visualization.

### Core genome phylogeny

Based on a 2.56 Mb core genome alignment we constructed a bootstrapped phylogenetic tree for the 38 *V *. *aestuarianus* isolates using RAxML-NG version 0.9.0 [29] (tree building parameters: --model GTR+G seed 2 --tree pars{25},rand{25}, bootstrapping parameters: --model GTR+G --bs-trees 200). For the larger *V. splendidus* dataset, we constructed a neighbour-joining tree using RapidNJ (parameters: --bootstrap 100) [30] with a core genome of 2.97 Mb. Each tree was rooted at the midpoint using Figtree [31]. Phylogenies were visualized using Microreact [32]. The project URLs are https://microreact.org/project/gfAsh7KuduL4xuSTDaVU5r-vibrio-aestuarianus (* V. aestuarianus *) and https://microreact.org/project/eMABqKLAPcn2QG5NEnCVor-vibrio-splendidus (* V. splendidus *). Pairwise SNP distances between isolates in the core genome alignment were calculated using Disty McMatrixface 0.1.0, which calculates pairwise differences in a given alignment, ignoring Ns in a pairwise manner [33].

### Phage prediction

We used PHAge Search Tool (PHAST) [34] to identify potential phages in clade A isolate 12 142, French clade B isolate 01 308 and Irish clade B isolate 16 060 as representative genomes for each genome condition. Fasta assembly files were assessed using default PHAST parameters.

All bioinformatics was carried out using resources provided by MRC-CLIMB [35].

## Results

### *V. aestuarianus*: presence of two clades in Ireland

**Fig. 2.**
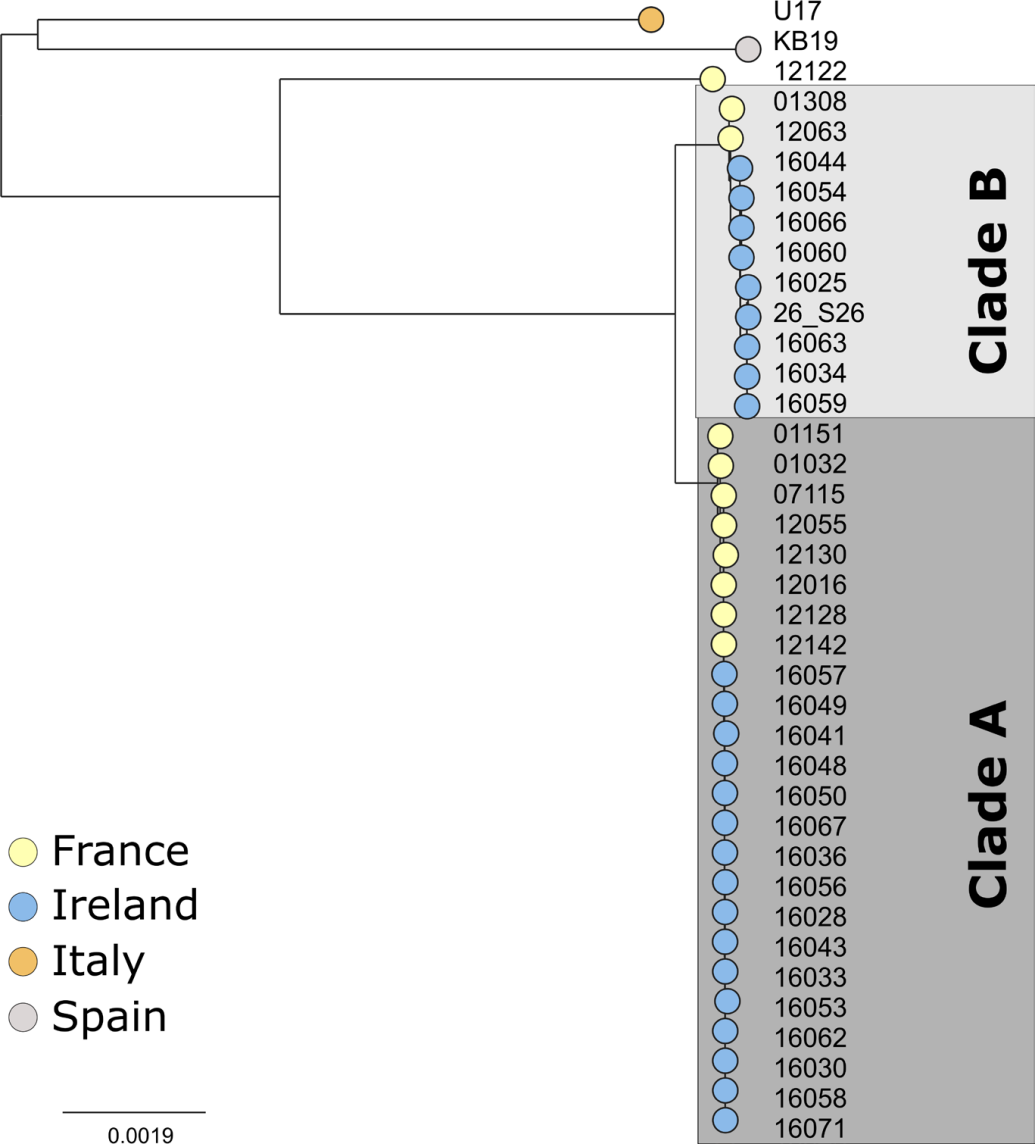
Core genome phylogeny of 38 *V *. *aestuarianus* isolates reveals two clades circulating in Ireland and France. A maximum-likelihood tree of 38 *V *. *aestuarianus* isolates constructed using a concatenated core genome alignment. The tree is rooted at the midpoint. The scale bar represents a mean of 0.0019 nucleotide substitutions per site. Tree tips are coloured by country of isolation. Isolates recovered in Ireland fall within two previously identified clades circulating in France, Clade A and clade B.

The core genome phylogeny of *V. aestuarianus* (Figs 2 and S2a) revealed that the French and Irish isolates were highly similar. The Irish isolates were resolved into the same two clades, A and B, previously reported to be circulating in French oyster culture [23]. Strains isolated from these two countries differ by 50 SNPs on average in clade A and 416 SNPs in clade B.

### *V. aestuarianus *: gene content variation in each clade

**Fig. 3.**
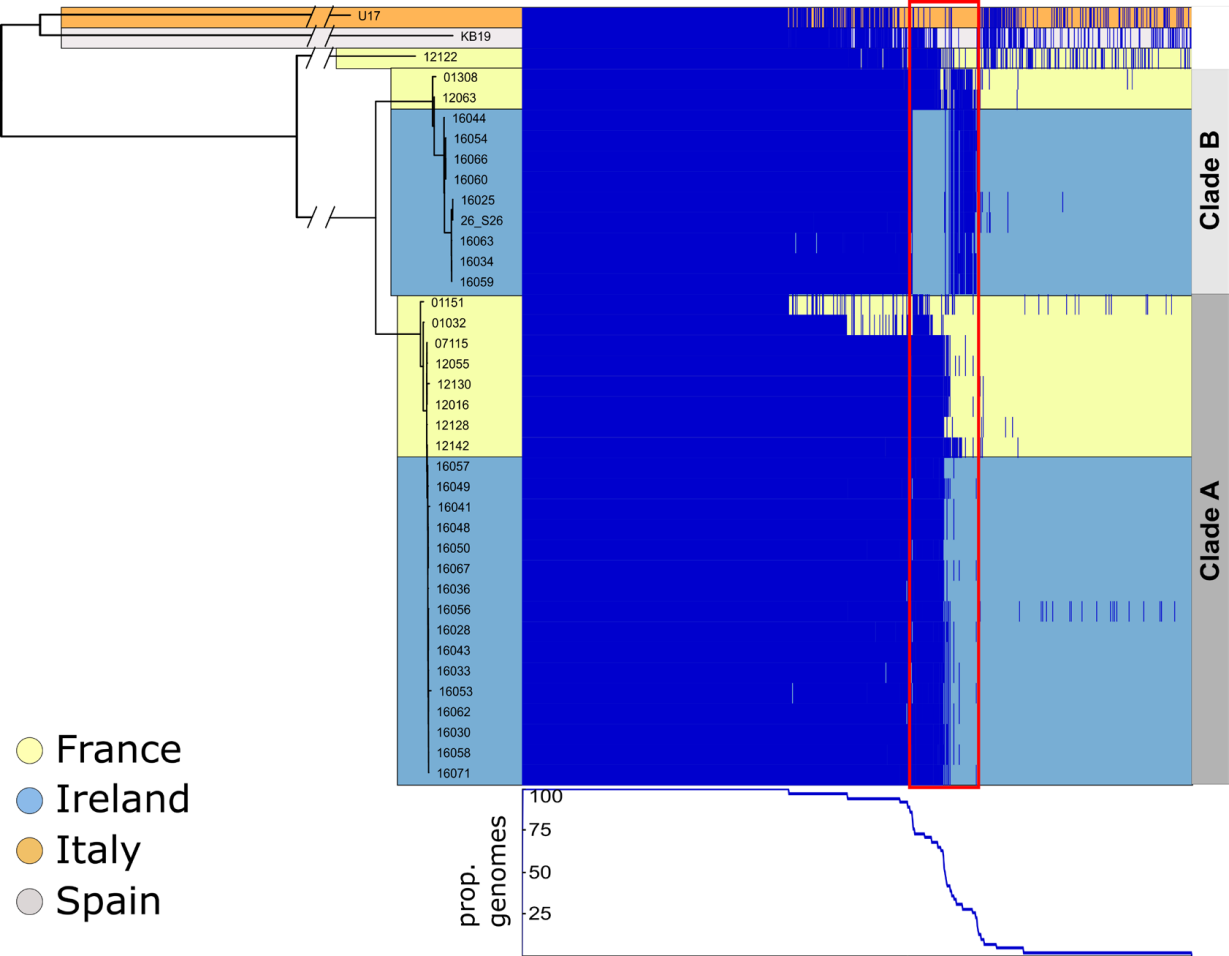
Gene presence–absence of 38 *V *. *aestuarianus *. Presence–absence heatmap of the pangenome of 38 *V *. *aestuarianus* genomes generated by Phandango [26]. Dark blue blocks indicate the presence of a gene family. The proportion of genomes each gene family has been detected in is shown below the heatmap. Tree branches and heatmap rows are coloured by country of isolation. Tree lengths have been truncated, see Fig. 2 for true branch lengths. Indicated in a red box are multiple genes that differ between clade A and clade B. Isolates 01151 and 01032, French clade B isolates, notably contain most of these genes.

The pangenome of *V. aestuarianus* consists of 5650 gene families (Fig. 3). This includes 2746 core gene families present in at least 95 % of isolates, 1150 shared by 10–95% isolates and 1754 shared by a single isolate up to 10 % of isolates. Isolates 01151 and 01032 are missing many core genes due to poor-quality assemblies: these were excluded from further pangenome analyses.

A set of 215 gene families present in all clade A isolates are absent in clade B isolates (Fig. 3). These genes are likely to have been horizontally acquired as mobile genetic elements (MGEs). To examine this, we checked the locations of these genes on the genome of the clade A isolate 12 142 and compared the GC content of these genes to the rest of the genome. The 215 genes resolved into 19 contiguous blocks of genes, each containing at least 2 genes (Table S3). The largest of these regions of contiguous genes contains 48 genes and has a GC content of 45.56 %, slightly higher than the genome average of 42.65 %. Another 13.5 kb region with 15 genes and a GC content of 43 % can be found 866 kb away from this region on the same contig. These two large gene regions have been identified as phages using PHAST (Table S4). The remaining contiguous blocks of genes are distributed across 11 contigs and contain mostly hypothetical proteins (108 of 130 genes). The presence of antitoxin- and phage-related proteins (YafN and IntA) suggests that many of these genes may lie on other uncharacterized mobile elements or plasmids.

Clade B isolates contain 92 gene families that are not shared with clade A, and the location of these genes was checked in clade B using isolate 16 060 as a representative genome. These are also largely hypothetical proteins (63 of 92) and are spread across 32 contigs in isolate 16 060, each carrying between 1 and 9 of these genes (Table S5). Genes related to two citrate fermentation operons that allow citrate to be used as an energy source in *V. cholerae *, *citCDEFXG* and *citS-oadGAB-citAB* [36], are only present in clade B isolates. *citD-G* and *citX* are all colocalized with *citB* and *citA* (also known as *dpiA* and *dpiB*). Genes *oadA*, *oadB* and *oadG* are found with *citC* and copies of *citA* and *citX*. No *citS* genes were detected in this species. Genes *citA* and *citG*, and one copy of *citX* are also found in one clade A genome: 12 142. *vspR*, a virulence gene repressor in *Vibrio cholera* [37], is also only found in Irish clade B genomes.

We also note that clade B strains isolated in France harbour both sets of genes, the 92 clade B genes and the 215 genes that are otherwise unique to clade A. This indicates that the clade B strains from Ireland included in this study have experienced extensive gene loss.

### *V. splendidus *: widespread clonal group uncovered

**Fig. 4.**
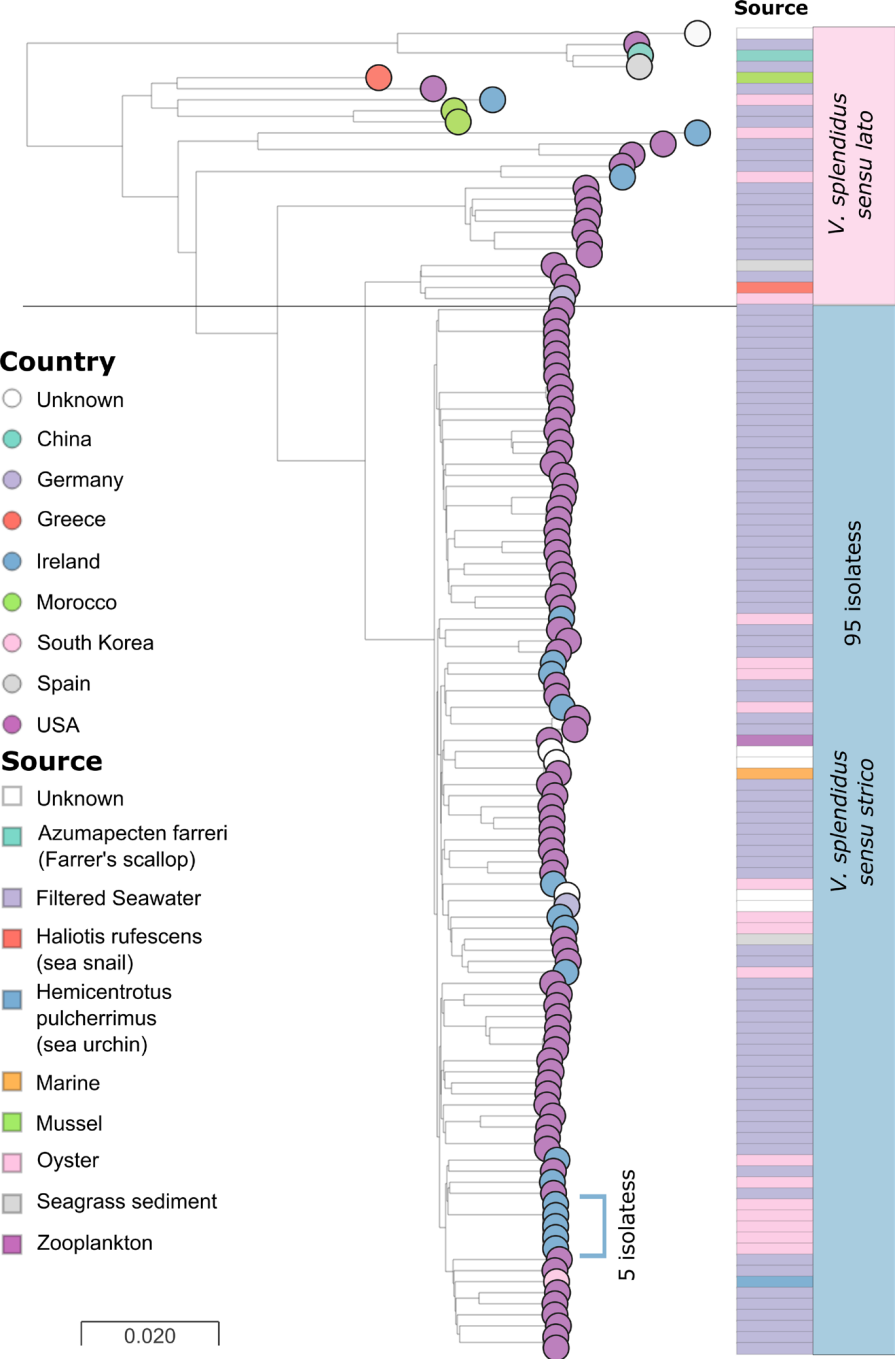
*
V. splendidus* core genome phylogeny. Neighbour-joining core genome phylogeny of 18 *V. splendidus* isolates sequenced here and 102 *V *. *splendidus* isolates accessed on the NCBI coloured by country of isolation. The scale bar represents an estimated dissimilarity of 2 % of the alignment. The tree is annotated with the source of isolation. Publicly available samples largely come from the USA and were sampled in seawater. The population structure of the dataset includes a large cluster of 95 genomes (purple). Isolates from Ireland are distributed throughout this population. However, one cluster of five highly similar isolates can be identified (blue).

**Fig. 5.**
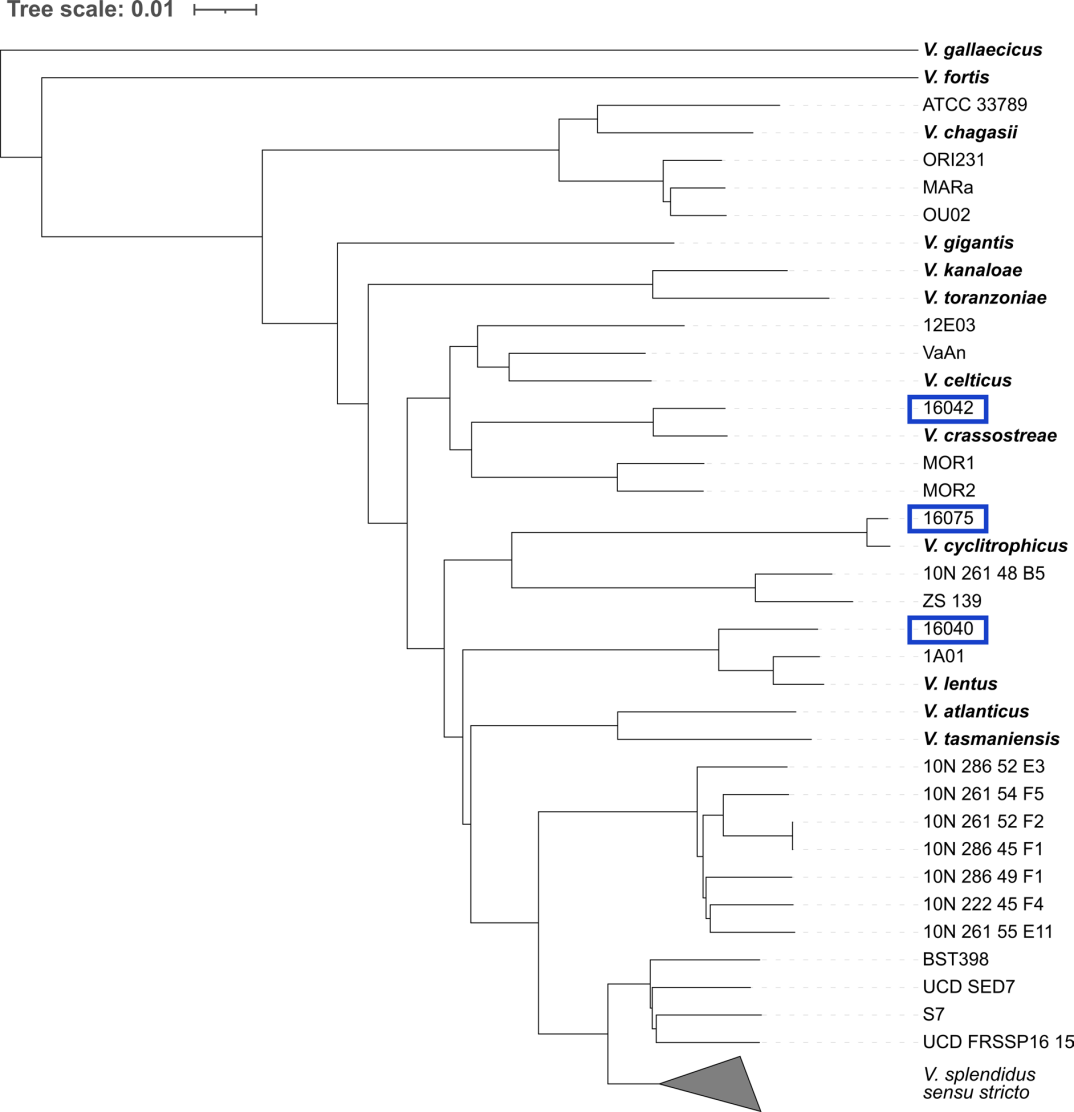
Phylogeny of *V. splendidus* species complex. A reference genome (in bold) for 13 species belonging to the *V. splendidus* species complex were combined with the 120 *V *. *splendidus* genomes used previously. Above is a neighbour-joining tree constructed using a core genome alignment. *V. splendidus* sensu stricto, containing a *V. splendidus* reference strain, is collapsed, and represents 96 isolates. Eleven of the publicly accessible genomes identified as *V. splendidus* species on NCBI that do not fall within *V. splendidus* sensu stricto are more similar to *V. splendidus *-like reference genomes. Similarly, three genomes isolated in Ireland – 16075, 16 040 and 16 042 seen in blue – are not found within the *V. splendidus* sensu stricto clade and are likely *V. splendidus *-like species.

**Fig. 6.**
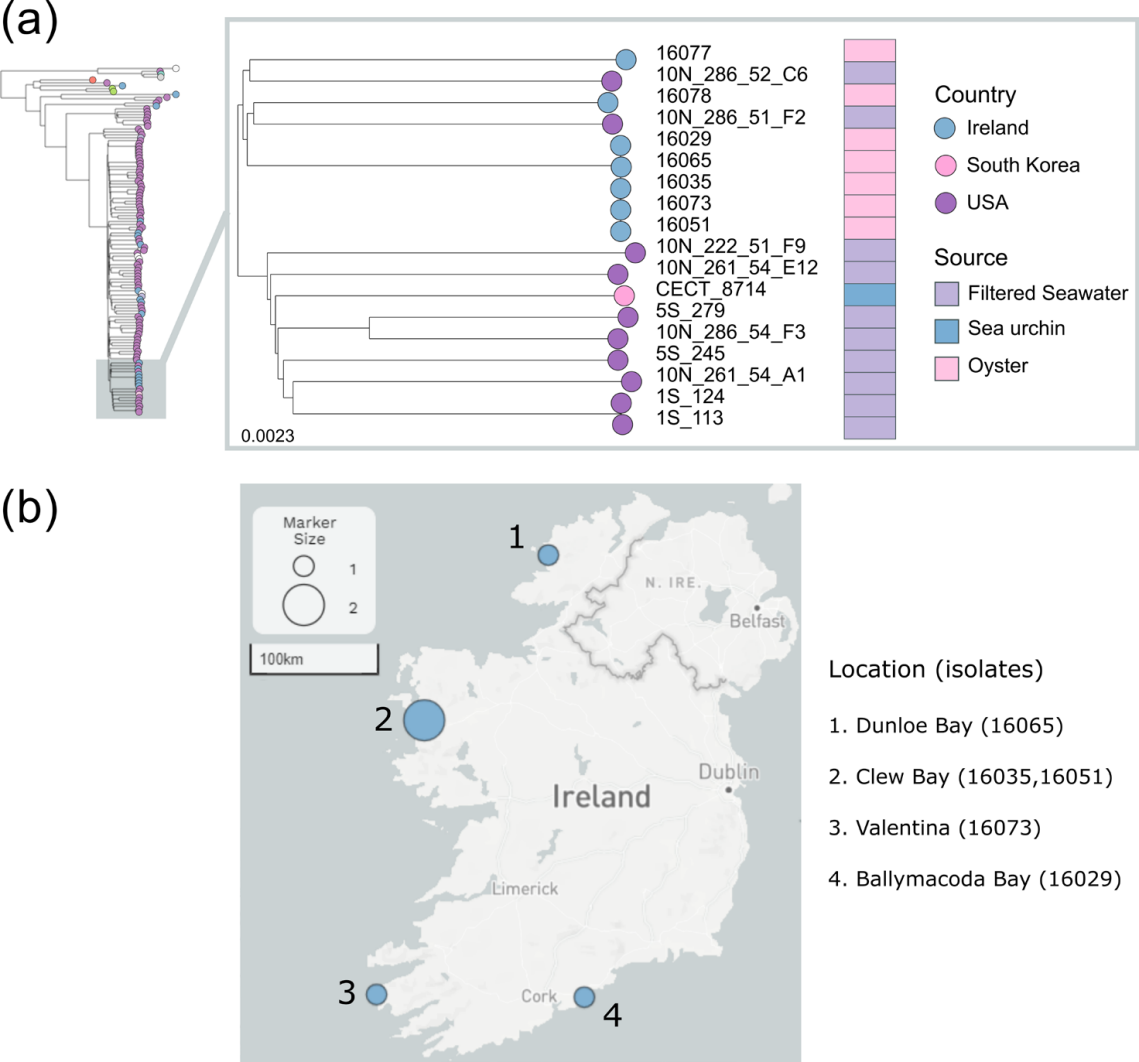
Subtree of *V. splendidus* reveals a cluster of five highly similar isolates in Ireland. (**a)** A subtree of 19 *V *. *splendidus* isolates from the tree shown in Fig. 4, including 5 Irish isolates with high similarity. Tree tips are coloured by country and annotated with the source of isolation. (**b)** Map of the five related *V. splendidus* strains shows these isolates were not recovered in the same locations. Nodes are weighted by the number of isolates per location.

To place *V. splendidus* isolates appearing in Irish oysters within the population structure of this species, we compared these 18 strains to 102 publicly available *V *. *splendidus* genomes. The phylogeny of *V. splendidus* isolates revealed a large cluster of 95 isolates, accompanied by multiple more diverse lineages (Figs 4 and S2b). Here we have referred to the large clade of 95 genomes as *V. splendidus* sensu stricto*,* while more diverse lineages are referred to as *V. splendidus* sensu lato. Of the newly sequenced strains, 15 are found within *V. splendidus* sensu stricto, while three strains lie within the broader population. Although the publicly accessed genomes were all classified as *V. splendidus* species, a phylogenetic comparison with reference genomes within *V. splendidus* clade has shown that many of the more diverse *V. splendidus* sensu lato isolates in this dataset are likely to have been misclassified (Fig. 5). Instead, these isolates are expected to represent other species from the *V. splendidus* species complex. Thus, we have designated isolates 16 040, 16 042 and 16 075 as *V. splendidus *-like isolates [38]. A cluster of five isolates recovered from four separate locations in Ireland show high similarity within this population (Fig. 6). These isolates differ by 28 SNPs on average across the core genome alignment, whereas the remaining 10 Irish isolates within the *V. splendidus* sensu stricto cluster differ by 83, on average.

The pangenome of this dataset contains 18 891 gene families, with a core genome of 3513 genes (95–100 % of isolates) and 13 270 rare accessory genes (0–10 % of isolates) (Fig. S1). Forty-two gene families are unique to the five Irish clonal group isolates. These include 18 genes dispersed within a 35.6 kb region, including a trio of resistance-related genes: cobalt–zinc–cadmium resistance protein, *czcA*; multidrug resistance protein, *mdtA*; and outer membrane protein *oprM*. Multiple genes related to stress response and signalling are also found in this region, including *nreB* oxygen sensor histidine kinase; *cmpR* a transcriptional activator involved in CO2 stress [39]; *htpG* a chaperone protein involved in general stress responses [40]; a putative signalling protein; and *pdeB*, a gene implicated in biofilm formation [41].

## Discussion

In Ireland, *V. aestuarianus* has been detected in oyster mortality events reported to the Marine Institute in 2001, 2003, 2006 and 2007, and more frequently in mortality events in spat from 2008 onwards, which had previously been attributed to OsHV-1 [42] (D. Cheslett, personal communication). Whilst mortality in adult oysters was only infrequently reported in Ireland prior to 2012, the frequency of reports and the detection of *V. aestuarianus* increased in line with those seen in France, particularly from 2015 onwards, following massive mortality events countrywide in 2015 [42, 43]. The trend of increased detections mirrored that in France; although the timeline of increased detections was later than that reported in France [11–13, 43]. The predominant pathogen detected in cases of adult and half-grown mortality in Ireland was *V. aestuarianus *, whilst that in spat was OsHV-1µVar. However, other bacteria, particularly other *Vibrio* sp., have also been isolated, mainly in conjunction with OsHV-1 and *V. aestuarianus *. Here by applying whole-genome sequencing we have characterized *Vibrio* strains that might play a major role in Irish oyster mortality events.

### Two *V. aestuarianus* clades linked with oyster mortalities in both Ireland and France

Our results show that all *V. aestuarianu* s strains detected in oysters in Ireland are members of two *V. aestuarianus* subsp. *francensis* clades, A and B, which have previously been detected in France [23]. SNP analysis revealed a high level of identity between the Irish and French *V. aestuarianus* isolates, suggesting that the clades causing disease outbreaks in France are also responsible for disease outbreaks across Ireland. There is a significant trade in live oysters between France and Ireland [12, 44], which has likely facilitated the movement of pathogens between rearing areas. However, broader genomic surveillance of *V. aestuarianus* associated with oyster mortalities is needed to uncover the exact distribution of each clade outside of these key *M. gigas*-producing countries.

A recent study involving the sequencing of *V. aestuarianus* strains across Europe showed that these two oyster-associated clades have now been found in multiple countries within Europe [45]. Their findings suggest that the emergence of these clades is the result of adaptation to a new environmental niche within which they have become oyster-specialist pathogens. The authors found low genomic diversity within each clade. Thus, the high genetic identity between Irish and French strains does not necessarily indicate a direct transmission chain between these two countries. While the data assessed here cannot be used to evaluate fine-scale transmission events between Ireland and France in *V. aestuarianu* s, we advocate for further whole-genome sequencing efforts within and across interconnected oyster-producing countries in Europe and elsewhere to help capture the spread and evolution of these emerging infectious clades [46].

### Evidence of gene loss in Irish clade B strains

Our data revealed a large number of gene families that are found in French but not Irish clade B isolates. This difference in genome content may suggest that a clade B strain was introduced once to Ireland, and that the founder population lost or previously lacked those genes. Although some of these genes were revealed to be on phages, the mechanisms of gene loss of the remaining 152 non-consecutive gene families in these otherwise highly related strains has not been determined. It is possible that this rapid genome reduction may have conferred a selective advantage to the Irish strains [47]. Given that these Irish strains are only compared to two strains from France, more extensive sequencing of clade B isolates across a wider range of affected regions is needed to evaluate the full diversity of the clade and determine whether this gene loss is exclusive to these Irish strains.

### A single clone of *V. splendidus* highlights transmission potential

*V. splendidus* clade strains were frequently detected in Irish oyster mortalities, although the role they played in disease is uncertain. Here we showed that these isolates were mostly distinct strains within a highly diverse species complex. *V. splendidus* is a highly diverse species and opportunistic pathogen [48, 49]. Given this, we would expect isolates associated with disease in Ireland to be largely unrelated, unless they happened to be isolated in the same location at one time or had recently been introduced through a common source. In 2009, a clonal group of highly similar isolates was found in multiple locations across Ireland (Fig. 6). In all cases, samples were taken where mortality was occurring in recently introduced French oyster seed. Both OsHV-1 µVar and *V. splendidus* were detected, suggesting that these isolates may be linked through the source of oyster seed. While this clonal group may have proliferated across Irish waters in 2009, given that such events have not been described in this species to date, it is much more likely that it was spread to multiple farms through a common source. Indeed, at least four of these isolates were found in sites which at that time contained stock from the same hatchery in France. The occurrence of this highly related clonal group of *V. splendidus* across multiple sites in the same year signifies the presence of transmission routes available to important oyster pathogens between production facilities.

### Perspectives

Pacific oyster summer mortality events in Ireland are shown here to be associated with two *V. aestuarianus* clades and a variety of strains within the *V. splendidus* complex. Notably, the two *V. aestuarianus* clades in Ireland have been described elsewhere in Europe, as clade A and B [23, 45]. Novel lineages were not detected, which underscores the importance of these two clades in Pacific oyster summer mortalities. The occurrence of a probable transmission event of *V. splendidus* across Ireland emphasizes the capacity for the spread of potentially pathogenic *Vibrio* within the oyster industry. Further genomic surveillance studies, which can build on this one, are needed within countries experiencing summer mortality syndrome and countries with which they frequently trade. This could lead to a fuller picture of the proliferation and evolution of this emerging pathogen worldwide and to better measures to prevent or deal with its future spread.

## Supplementary Data

Supplementary material 1

Supplementary material 2
