# Supplementary material for: Vibrio aestuarianus clade A and clade B isolates are associated with Pacific oyster (Magallana gigas) disease outbreaks across Ireland
Source: Microb Genom. 2023 Aug 4;9(8):mgen001078. doi: 10.1099/mgen.0.001078 (PMC10483421; doi:10.1099/mgen.0.001078)
Supplement: Supplementary material 1 [file mgen-9-1078-s001.pdf]

Supplementary materials for "*Vibrio aestuarianus* Clade A and Clade B isolates are associated with Pacific oyster (*Magallana gigas*) disease outbreaks across Ireland"

Supplementary tables:

Table S1: Data source information and quality control values for sequencing data used in this study.

Table S2: Metadata for publicly available data.

Table S3: Gene families unique to Clade A isolates as seen in genome 12142. Genes are displayed in order of appearance in each contig. Nineteen co-localised blocks of gene families are shown.

Table S4: PHAST results for genome 12142.

Table S5: Gene families unique to Clade B isolates as seen in genome 16060. Genes are displayed in order of appearance in each contig. Twenty-two co-localised blocks of gene families are shown.

Supplementary figures:

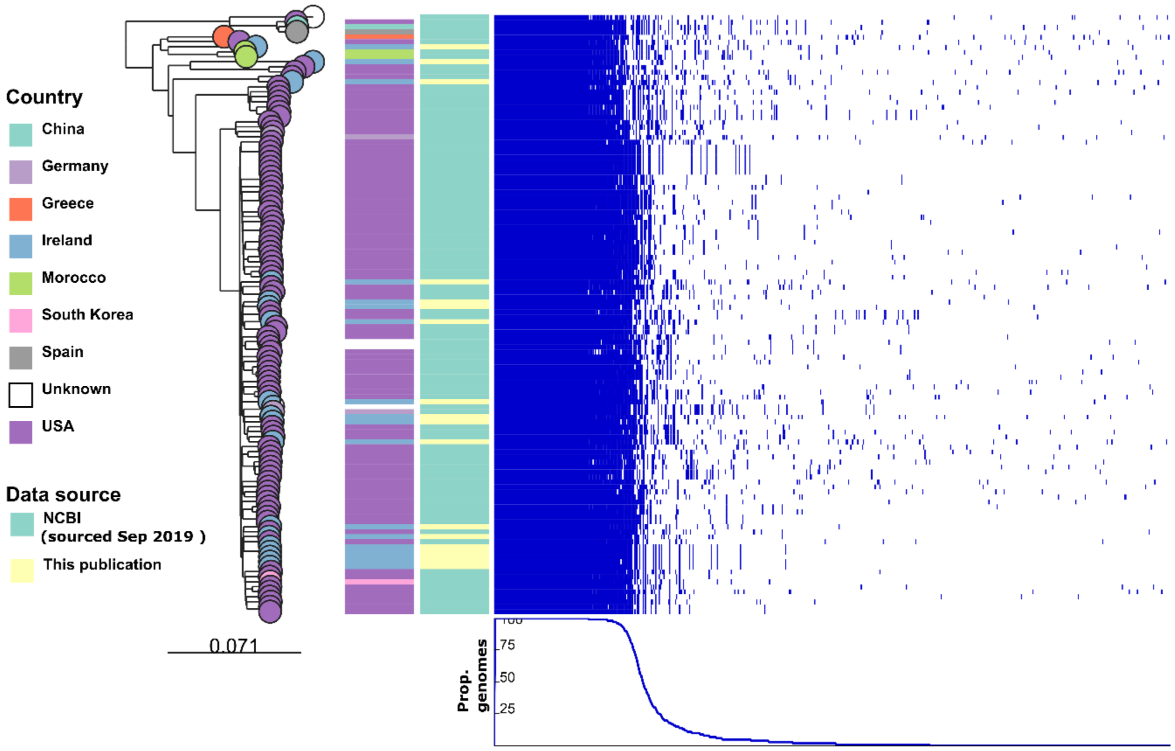

Figure S1: *V. splendidus* gene family presence-absence in 120 isolates. Gene presence-absence for 18 *V. splendidus* isolates and 102 isolates identified as *V. splendidus* on the NCBI. The country of isolation is indicated in the tree leaves and the first metadata column. A second column indicates the Data source of each isolate. A heatmap is shown alongside the tree where dark blue indicates the presence of a gene family in a genome. The heatmap is ordered by the proportion of genomes containing each gene family which is plotted underneath.

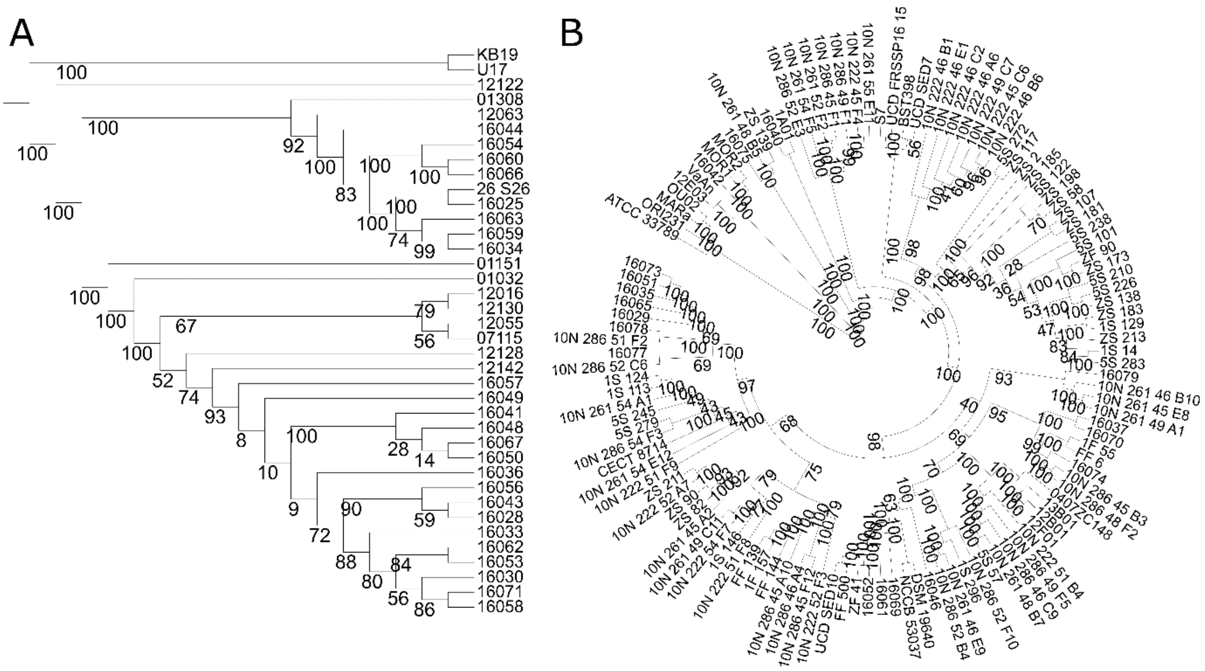

Figure S2: Phylogenetic trees with bootstrap values added. A) An ML tree of 38 *V. aestuarianus* isolates as shown in Figure 2 with branch lengths removed and bootstrap values added. B) A Neighbour-Joining tree of 120 *V. splendidus* species complex isolates as shown in Figure 4 with branch lengths removed and bootstrap values added.
